# G6PDH activity highlights the operation of the cyclic electron flow around PSI in *Physcomitrella patens* during salt stress

**DOI:** 10.1038/srep21245

**Published:** 2016-02-18

**Authors:** Shan Gao, Zhenbing Zheng, Li Huan, Guangce Wang

**Affiliations:** 1 Key Laboratory of Experimental Marine Biology, Institute of Oceanology, Chinese Academy of Sciences, Qingdao 266071, China; 2 Laboratory for Marine Biology and Biotechnology, Qingdao National Laboratory for Marine Science and Technology, Qingdao, China; 3 University of Chinese Academy of Sciences, Beijing, 100049, China

## Abstract

Photosynthetic performances and glucose-6-phosphate dehydrogenase (G6PDH) activity in *Physcomitrella patens* changed greatly during salt stress and recovery. In *P. patens*, the cyclic electron flow around photosystem (PS) I was much more tolerant to high salt stress than PSII. After high salt stress, the PSII activity recovered much more slowly than that of PSI, which was rapidly restored to pretreatment levels even as PSII was almost inactivate. This result suggested that after salt stress the recovery of the cyclic electron flow around PSI was independent of PSII activity. In addition, G6PDH activity and NADPH content increased under high salt stress. When G6PDH activity was inhibited by glucosamine (Glucm, a G6PDH inhibitor), the cyclic electron flow around PSI and the NADPH content decreased significantly. Additionally, after recovery in liquid medium containing Glucm, the PSI activity was much lower than in liquid medium without Glucm. These results suggested the PSI activity was affected significantly by G6PDH activity and the NADPH content. Based on the above results, we propose that G6PDH in *P. patens* has a close relationship with the photosynthetic process, possibly providing NADPH for the operation of the cyclic electron flow around PSI during salt stress and promoting the restoration of PSI.

The moss *Physcomitrella patens* has a relatively simple morphology, and its gametophyte is dominant in the life cycle. Moreover, it is a multicellular eukaryote that demonstrates a high rate of homologous recombination^1^. These advantages make it an ideal model for the analysis of most aspects of plant biology. More importantly, *P. patens* is highly tolerant to a variety of stresses, such as cold, drought and high salt^2^,^3^,^4^,^5^,^6^. As a result, it has been used in investigating the responses of plants to abiotic stresses.

The abiotic stresses, such as drought and high salt, profoundly affect the physiological functions of plants^7^,^8^. In fact, a number of physiological processes, including photosynthesis and carbohydrate metabolisms, are sensitive to abiotic stresses. Photosynthesis, and especially photosynthetic electron flow, is a sensitive sensor for different stresses, which not only provides energy but also represents the reception of environmental information^9^,^10^,^11^. Many studies have reported that photosynthetic electron flow, particularly the cyclic electron flow around PSI, plays a significant physiological role in plant responses to stresses, which could provide ATP and protect photosynthetic apparatus under stress conditions^12^,^13^,^14^,^15^,^16^,^17^. In addition, during re-hydration, the recovery of PSI in desiccated macro-algae was much faster than that of PSII, and could still be restored when PSII was suppressed^18^,^19^. This introduces the issue of the source of the electrons involved in the cyclic electron flow around PSI during recovery when PSII is inhibited.

When plants are subjected to stresses, not only does the photosynthetic process change significantly, but also the carbohydrate metabolism, particularly the oxidative pentose-phosphate pathway (OPPP), demonstrates a positive response to stresses. Glucose-6-phosphate dehydrogenase (G6PDH), which catalyzes the first step of the OPPP and regulates NADPH provision in plants, is a key enzyme of the OPPP. The activity and content of G6PDH rise remarkably in stressed plants^20^,^21^,^22^,^23^. NADPH is an important molecule in the redox balance of plant cells and is also required for plant protection against oxidative damage because many environmental conditions induce oxidative stress. In addition, NADPH can donate electrons to the photosynthetic electron flow^12^,^24^. This also raises the question of whether the OPPP provides NADPH for the operation of the cyclic electron flow around PSI during recovery.

The aim of this work was to study the responses of the photosynthetic electron flow and G6PDH in *P. patens* to high salt stress and the physiological link between the two processes. Particular attention was paid to the restoration of PSI and PSII during recovery and the responses of G6PDH activity and NADPH content to salt stress. The data obtained demonstrate that G6PDH has a close relationship with the photosynthetic process and might provide NADPH for the operation of the photosynthetic electron flow and the promotion of PSI restoration.

## Results

### The photosynthetic activities of gametophores in response to salt stress

**Figure 1:**
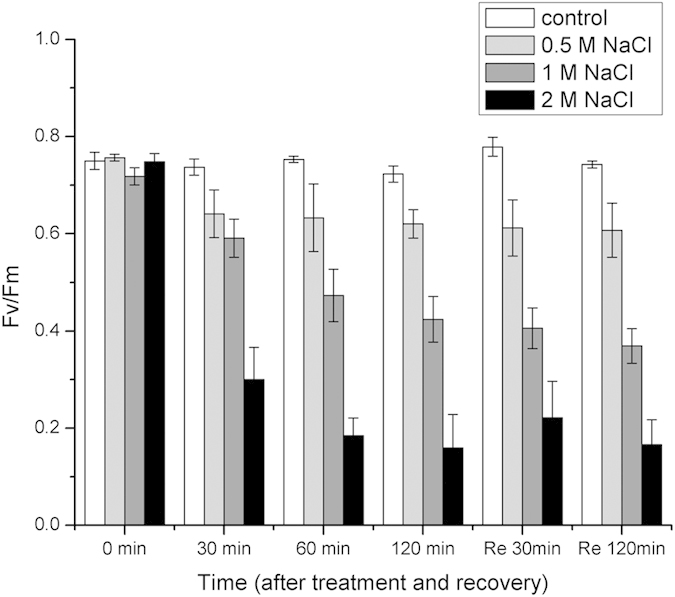
Changes in F_v_/F_m_ in *P. patens* during treatments with control (normal liquid medium), 0.5 M, 1 M and 2 M NaCl solutions and after recovering in normal liquid medium. Re is the abbreviation of recovery. Data shown are the means of five independent experiments (±SD).

**Figure 2:**
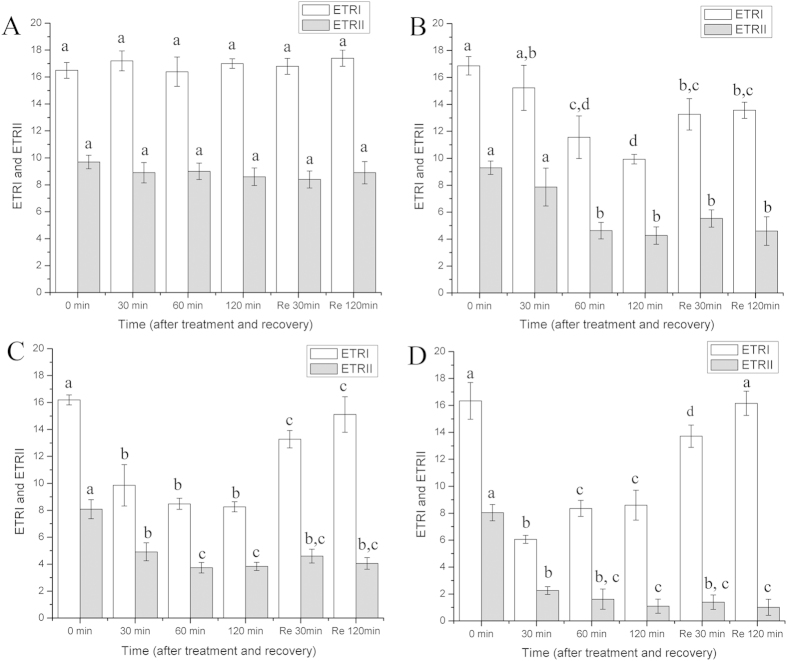
Variations in ETRII and ETRI in *P. patens* during treatments with control (normal liquid medium) (A), 0.5 M (B), 1 M (C) and 2 M (D) NaCl solutions and after recovering in normal liquid medium. Re is the abbreviation of recovery. Different letters represent significant differences between different times of salt treatment (p < 0.05, ANOVA, followed by Tukey’s post-hoc test for comparisons, α = 0.05). Data shown are the means of five independent experiments (±SD).

Salt stress has great impact on the photosynthetic activities of the gametophores (Figs 1 and 2, and Supplementary Figure S1). The maximum quantum yield (F_v_/F_m_) decreased slightly during the course of the 0.5 M NaCl treatments in comparison with the control. After recovering in liquid medium for 30 min and 120 min, the F_v_/F_m_ did not change significantly. In contrast, when the salinity increased (1 M and 2 M NaCl), F_v_/F_m_ declined significantly and remained at a low level during recovery (Fig. 1). In comparison with the control (Fig. 2A), the activities of PSII and PSI decreased gradually during 0.5 M NaCl treatments, as suggested by the ETRII and ETRI. After recovery, both PSII and PSI could be restored (Fig. 2B). During the high salt treatments (1 M and 2 M NaCl), the PSII activity declined dramatically and could not be restored after 120 min of recovery. After 30 min of treatment with high salt solution, the PSI activity decreased significantly; however, with more prolonged treatments, the PSI activity did not change and was maintained at a low level (Fig. 2C,D). More importantly, after 30 min of recovery in liquid medium, even though the PSII activity was not restored, the PSI activity was rapidly restored, and after 120 min of recovery, it was almost restored to the pretreatment level. This phenomenon was much clearer in the gametophores treated with 2 M NaCl (Fig. 2D) than those treated with 1 M NaCl (Fig. 2C). The results suggested that the PSI in the gametophores of *P. patens* demonstrated a higher degree of tolerance to high salt stress than PSII and after treatments with high salt solution, the recovery of PSI activity was independent of the recovery of PSII activity.

### Effects of G6PDH activity on the recovery of photosynthetic performance

**Figure 3:**
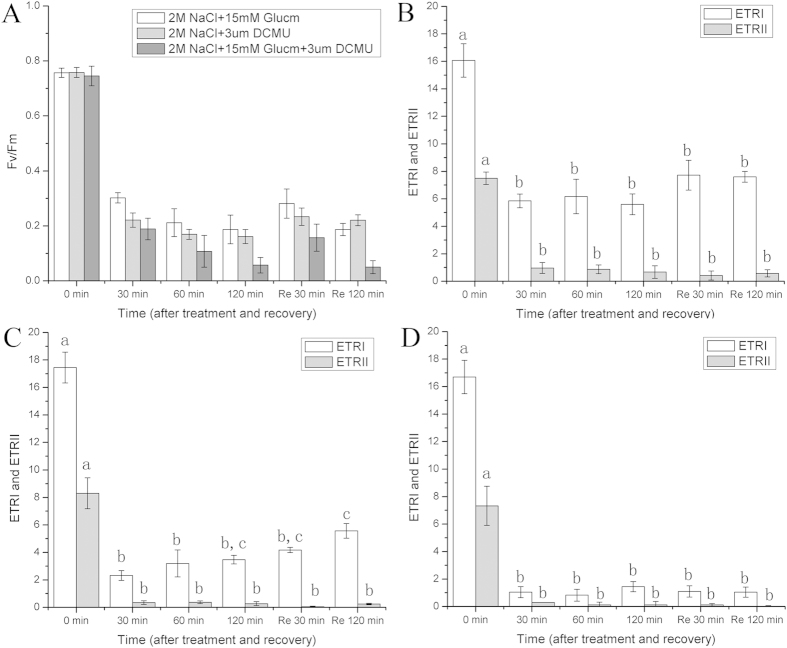
Responses of F_v_/F_m_, ETRII and ETRI in *P. patens* to 2 M NaCl solution containing DCMU and Glucm. (**A**) Changes in F_v_/F_m_ in *P. patens* during immersion in 2 M NaCl solutions which contained 15 mM Glucm, 3 uM DCMU and the two inhibitors, respectively; Responses of ETRII and ETRI in *P. patens* during immersion in 2 M NaCl solution to 15 mM Glucm (**B**), 3 uM DCMU (**C**) and the two inhibitors (**D**). Re 30 min and Re 120 min means that the gametophores were restored in liquid medium containing the same inhibitor as they were treated with 2 M NaCl solutions. Different letters represent significant differences between different times of salt treatment (p < 0.05, ANOVA, followed by Tukey’s post-hoc test for comparisons, α = 0.05). Data shown are the means of five independent experiments (±SD).

The PSII and PSI activities during the course of high salt stress (2 M NaCl) were affected significantly by different inhibitors (Fig. 3). Glucm could effectively inhibit the G6PDH activity, which is the key enzyme that catalyzes the first step of the OPPP to produce NADPH. When the high salt solution contained Glucm, the PSI activity decreased significantly (p < 0.05), especially with prolonged treatment (Fig.3B). More importantly, after recovery in liquid medium containing Glucm, the ETRI (around 7) was much lower than the ETRI in liquid medium without Glucm (around 16) (Fig. 2D), suggesting that the PSI activity was affected significantly by the G6PDH activity. Meanwhile, the ETRII declined slightly in comparison with ETRII in the no Glucm condition. In contrast, when the high salt solution contained DCMU (a PSII inhibitor), both ETRI and ETRII decreased significantly (p < 0.05)(Fig. 3C). After recovering in liquid medium containing DCMU, ETRI was even lower than in the gametophores treated with Glucm. Furthermore, when the gametophores were treated with a high salt solution containing the two inhibitors (Fig. 3D), ETRII and especially ETRI decreased to very low levels. These results suggested that PSII and G6PDH activities during the course of high salt stress play important roles in the recovery of PSI activity.

**Figure 4:**
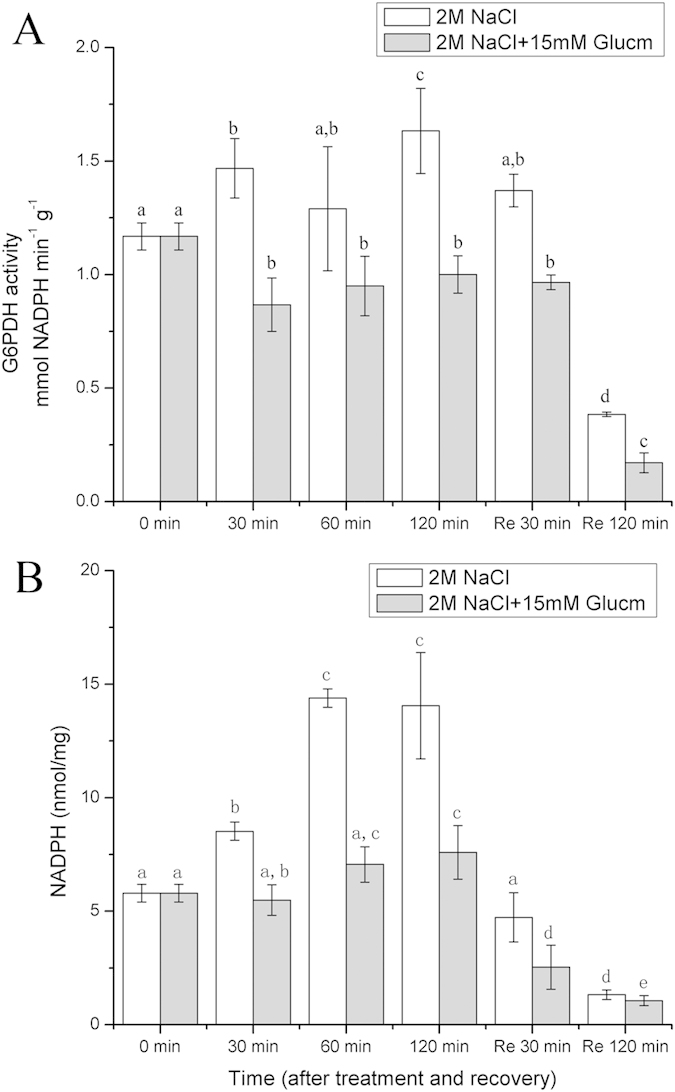
Effects of 2 M NaCl solution on G6PDH activity and NADPH content in *P. patens* and responses of G6PDH and NADPH to 15 mM Glucm. Re 30 min and Re 120 min means that the gametophores were restored in liquid medium.(**A**) G6PDH activity. (**B**) NADPH content. Different letters represent significant differences between different times of salt treatment (p < 0.05, ANOVA, followed by Tukey’s post-hoc test for comparisons, α = 0.05). Data shown are the means of five independent experiments (±SD).

We further determined the G6PDH activity and the NADPH content of the gametophores during the course of high salt stress. As Fig. 4 shows, the G6PDH activity was enhanced by the high salt stress. After 120 min of treatment, the G6PDH activity increased to the highest level (Fig. 4A). Nonetheless, during recovery, the G6PDH activity declined significantly (p < 0.05) and was maintained at a lower level. Meanwhile, the NADPH content increased greatly during the high salt treatment and rose to its highest value after 120 min of treatment, which was consistent with the G6PDH activity during high salt stress. In addition, when Glucm was present in the high salt solution, the G6PDH activity was inhibited greatly (Fig. 4A). During recovery in the liquid medium containing Glucm, the G6PDH activity was much lower than when in medium without Glucm. Moreover, the NADPH content decreased significantly (p < 0.05) with a prolonged treatment in the high salt solution containing Glucm, which was consistent with the G6PDH activity. These results further demonstrated that the G6PDH activity greatly affected the photosynthetic electron flow during high salt stress and especially the recovery of PSI activity.

### Changes in PRK activity and RNA content during high salt stress

**Figure 5:**
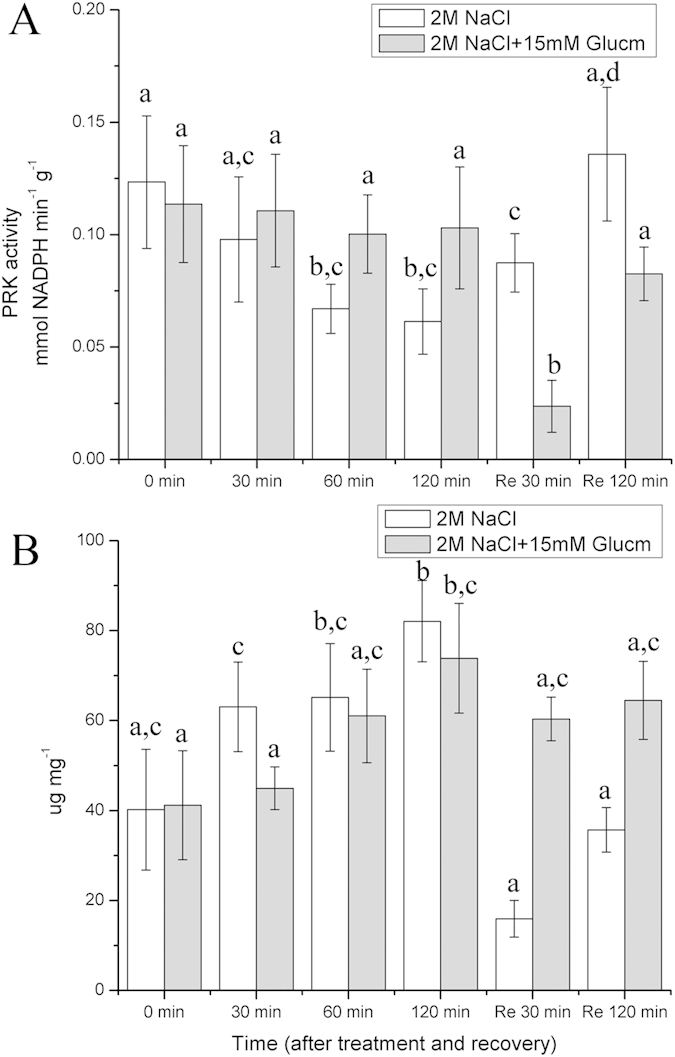
Effects of 2 M NaCl solution on PRK activity and RNA content in *P. patens* and responses of PRK and RNA to 15 mM Glucm. Re 30 min and Re 120 min means that the gametophores were restored in liquid medium. (**A**) PRK activity. (**B**) RNA content. Different letters represent significant differences between different times of salt treatment (p < 0.05, ANOVA, followed by Tukey’s post-hoc test for comparisons, α = 0.05). Data shown are the means of five independent experiments (±SD).

PRK, an enzyme involved in the Calvin cycle, decreased gradually during the course of the high salt stress. After 120 min of recovery, its activity had increased greatly. Moreover, during the high salt stress, there was a slight increase in the PRK activity in the presence of the G6PDH inhibitor (Glucm), although this was not significant (Fig. 5A). Additionally, after 120 min of treatment with the high salt solution, the RNA content increased significantly (p < 0.05). After recovery, the RNA content decreased dramatically. In the presence of Glucm, there was a small decrease during the course of the high salt treatment (Fig. 5B, Supplementary Figure S2).

### Starch and soluble sugar responses to salt stress

**Figure 6:**
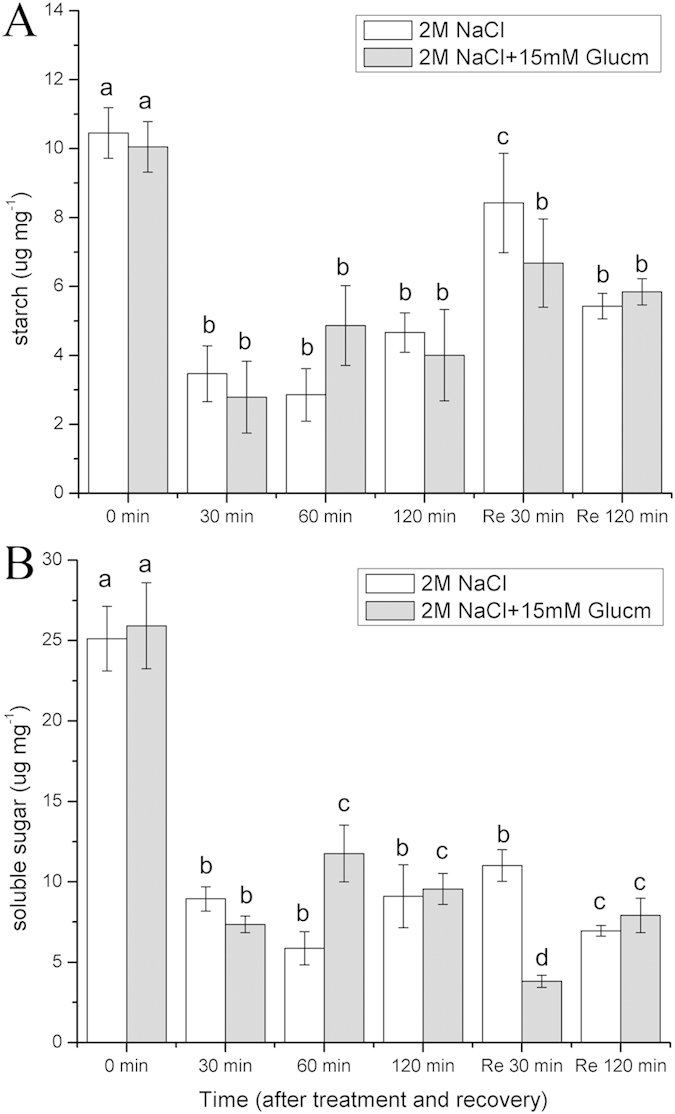
Effects of 2 M NaCl solution on starch and soluble sugar content in *P. patens* and responses of starch and soluble sugar levels to 15 mM Glucm. Re 30 min and Re 120 min means that the gametophores were restored in liquid medium. (**A**) starch content. (**B**) soluble sugar content. Different letters represent significant differences between different times of salt treatment (p < 0.05, ANOVA, followed by Tukey’s post-hoc test for comparisons, α = 0.05). Data shown are the means of five independent experiments (±SD).

Our results suggested that high salt stress greatly affects the starch and soluble sugar contents in the gametophores. The starch content decreased significantly (p < 0.05) during the course of the high salt treatment (Fig. 6A). When the gametophores recovered, the starch content increased dramatically. Moreover, Glucm barely affected the starch content of gametophores during high salt stress and recovery. Meanwhile, the soluble sugar content decreased significantly (p < 0.05) during the course of the high salt stress. After recovery, its content did not change significantly. In addition, Glucm had small effects on the soluble sugar content of the gametophores during high salt stress and recovery. In particular, after 30 min of recovery in the presence of Glucm, the sugar content declined significantly (p < 0.05) (Fig. 6B).

## Discussions

It is known that F_v_/F_m_ is a sensitive indicator of the photosynthetic performance of plants and decreases when plants are subjected to stresses^25^. Our results showed that F_v_/F_m_ declined substantially during salt stress (Fig. 1), demonstrating that the photosynthetic performance of *P. patens* was affected greatly by salt stress. During high salt treatment, ETRII decreased to a very low level (almost to 0) whereas the ETRI value remained high, suggesting that the PSI in *P. patens* was much more tolerant to salt stress than the PSII. In fact, similar phenomena occur in other drought-tolerant plants and algae, such as *Paraboea rufescens*, *Ulva prolifera* and *Porphyra yezoensis*^16^,^18^,^19^. Actually, both salt stress and drought could induce dehydration (osmotic stress) of plants, but salt stress also comprises ionic stress since the inevitable uptake or loss of ions will cause ionic stress to plants. Therefore, although the PSI in plants demonstrated much more tolerant to drought and salt stress, the response mechanisms to the two different stresses may be different^5^,^8^. In particular, the recovery of the PSI activity was much faster than that of PSII (Fig. 2B,C,D). Moreover, when PSII was inhibited by DCMU, PSI could still be restored, indicating that the recovery of the cyclic electron flow around PSI was independent of PSII activity after salt stress. It has been reported that in some intertidal macro-algae, after recovering from stresses, even though the linear electron flow was suppressed by DCMU, the cyclic electron flow around PSI could still be restored^18^,^23^. Based on the above results and the published data, we hypothesized that there may be other electron sources (in addition to electrons from water oxidation at PSII) that could donate electrons to PSI under salt stress and especially during recovery.

Many studies have reported that G6PDH plays an important role in the adaptation to a variety of stresses, such as salt stress and drought^20^,^22^,^26^. Under salt stress or drought conditions, the G6PDH activity in soybean roots was greatly enhanced, which modulated redox homeostasis under environmental stresses 21,27. Both the G6PDH activity and the NADPH content in the stress-tolerant green macro-algae *U. prolifera* increased significantly during salt stress 23. In addition, Rai, *et al.*^28^ reported that the NADPH level was induced in *Anabaena* cells under salt stress, and the induction of NADPH and transketolase indicated OPPP’s operation during salt stress. In fact, G6PDH is the key regulatory enzyme of the OPPP, which controls the flow of carbon and produces NADPH to meet the cellular needs of reductive biosynthesis^29^. Our results suggested that during the course of salt stress, the G6PDH activity in *P. patens* increased significantly (p < 0.05) (Fig. 4A), which was consistent with the changes in the NADPH levels (Fig. 4B). Moreover, the cyclic electron flow around PSI could still operate during salt stress and be restored rapidly after recovery from salt stress when PSII was inactivated. In addition, as G6PDH activity was obviously inhibited by Glucm (a G6PDH inhibitor)^27^, both the cyclic electron flow around PSI and the NADPH content decreased significantly (p < 0.05), suggesting that there was a close relationship between them (Figs 3 and 4). In fact, NAD(P)H can donate electrons to photosynthetic electron carriers, which may have significant effects on the cyclic electron flow around PSI^24^,^30^. Based on our results and the published data, we proposed that the NADPH produced by G6PDH during the OPPP could provide electrons for the operation of the cyclic electron flow around PSI in *P. patens* during salt stress and recovery.

In addition to producing NADPH, the OPPP also provides R5P^31^. R5P not only participates in the Calvin cycle during the generation of ribulose 1,5-bisphosphate, but it can also be utilized for nucleotide synthesis^32^. The enzymes of the Calvin cycle are sensitive to stresses^33^. Furthermore, Huan, *et al.*^23^ reported that the PRK activity of the Calvin cycle in *U. prolifera* declined greatly under high salt stress, which catalyzes the conversion of R5P to ribulose 1,5-bisphosphate. Our results suggested that the PRK activity in *P. patens* decreased significantly (p < 0.05)under salt stress (Fig. 5A). Moreover, the RNA content increased significantly (p < 0.05) under salt stress (Fig. 5B, Supplementary Figure S2). A plausible explanation is that the increase of G6PDH activity and the decrease of PRK activity resulted in an accumulation of pentose, which is likely to improve RNA synthesis. In fact, it has been proposed that the excess synthesis of total RNA might help the cells overcome stress and ready themselves for non-stressed conditions^23^. Thus, we suggested that the increased RNA content in *P. patens* during salt stress increases the moss’ ability to survive salt stress.

In addition, the starch content in *P. patens* decreased significantly (p < 0.05) during salt stress, suggesting that salt stress accelerated starch degradation. In fact, a variety of stresses, including temperature and drought stress, could induce starch degradation^23^,^34^,^35^,^36^. Moreover, Johnson and Alric^24^ proposed that the starch breakdown plays an important role in the donation of electrons to the photosynthetic cyclic electron flow around PSI in *Chlamydomonas reinhardtii*. In addition, our results indicated that the soluble sugar content in *P. patens* decreased significantly (p < 0.05) during salt stress, demonstrating decomposition. Based on our results and the published data, we suggested that the increased starch degradation and G6PDH activity in *P. patens* during salt stress improved the generation of NADPH and pentose, which could donate electrons to the photosynthetic electron flow and be used during RNA synthesis, respectively.

## Materials and Methods

### Plant materials and growth conditions

*P. patens* ecotype ‘Gransden 2004’ (gift from professor Yikun He, Capital Normal University, China) was grown in BCD medium (1 mM MgSO_4_, 10 mM KNO_3_, 45 mM FeSO_4_, 1.8 mM KH_2_PO_4_, pH 6.5) containing 0.5% (w/v) Glc and 0.75% (w/v) agar 4. Gametophores were cultured at a light intensity of 60 μmol photons m^−2^ s^−1^ (16 h light/8 h dark). Four-week-old gametophores were used in the experiments.

### Salt stress treatment and inhibitor treatment

Four-week-old gametophores were treated with control (normal liquid medium), 0.5 M, 1 M and 2 M NaCl for 2 h, respectively. During the course of these treatments, the photosynthetic activities of the gametophores were measured after 30, 60 and 120 min. For recovery, the gametophores were transferred into liquid medium. During recovery, the photosynthetic activities of the gametophores were measured after 30 and 120 min.

To investigate the effects of PSII and GAPDH on the PSI activity under salt stress, the inhibitors 3-(3′,4′-dichlorophenyl)-1,1-dimethylurea (DCMU, a PSII inhibitor) and glucosamine (Glucm, a G6PDH inhibitor) were used in this study^19^,^27^. Four-week-old gametophores were treated with 2 M NaCl containing either 3 μm DCMU or 15 mM Glucm for 2 h. During treatment, the PSI and PSII activities were measured after 30, 60 and 120 min. Subsequently, the gametophores were transferred into liquid medium containing either 3 μm DCMU or 15 mM Glucm. After 30 and 120 min of recovery, the activities of PSI and PSII were measured. In addition, the activities of PSI and PSII were monitored under 2 M NaCl solution containing both 3 μm DCMU and 15 mM Glucm. Then, the gametophores were transferred into liquid medium containing both 3 μm DCMU and 15 mM Glucm for recovery. The activities of PSI and PSII were measured after 30 and 120 min of recovery.

### Chlorophyll fluorescence and P700 measurement

As described previously^18^, the chlorophyll fluorescence of PSII and the redox state of P700 (an indicator of PSI activity) of the gametophores were measured concomitantly during high salt treatment using a Dual-PAM-100 fluorometer (Walz, Effeltrich, Germany) connected to a computer. Before measurement, the gametophores were dark-adapted for 10 min. F_0_ (the minimum fluorescence) was determined and subsequently a saturating flash was applied to detect the maximal fluorescence (F_m_). The difference between F_m_ and F_0_ was referred to as the variable fluorescence (F_v_), and the maximum quantum yield was obtained as F_v_/F_m_^37^. F_m_’ was detected as the gametophores were illuminated. The steady-state value of fluorescence immediately before the saturating flash is termed F_t_. The effective photochemical quantum yield of PS II [Y(II)] is calculated as (F_m_’− F_t_)/ F_m_’. It is assumed that photons are evenly distributed between PSI and PSII and an average of leaf absorbs about 84% of incident PAR (photosynthetically active radiation)^37^,^38^. Hence, in Dual-PAM-100 system, the following expression serves for the relative electron transport rate (ETR): ETRII = 0.84 × 0.5 × PAR × Y(II) = 0.42 × PAR × Y(II)^37^,^38^.

In analogy to chlorophyll fluorescence measurement, the saturation pulse method was used to determined the P700 parameters^39^. Although the measurement of P700 was often affected by plastocyanin (PC) signal when the excitation wavelength is 700 nm, but in Dual-PAM-100 system, P700 was measured in the dual-wavelength mode (difference of intensities of 875 and 830 nm pulse modulated measuring light reaching the photodetector)^39^. The PC signal still exists in Dual-PAM-100 system its effect on P700 measurement was very weak^40^. The maximal P700 signal, P_m_, was determined by application of the saturation pulse in the presence of far-red light, which was defined in analogy to F_m_. The zero P700 signal (P_0_) was determined when complete reduction of P700 was induced after the saturation pulse and cessation of far-red illumination. The maximal P700 signal (P_m_’) was induced by combined actinic illumination with the saturation pulse. Based on P_m_, P_0_ and P_m_’, Y(I) was calculated automatically by Dual-PAM-100 software. Moreover, ETRI was calculated by Dual-PAM-100, which was defined in analogy to ETRII^41^.

### Determination of G6PDH and phosphate-5-ribulose kinase (PRK) activities

For the determination of G6PDH activity, an equal wet weight, 0.1 g, of gametophores selected at different times during the salt treatments was frozen in liquid nitrogen and ground into powder. Then, 300 μl extraction buffer, containing 50 mM Hepes-Tris (pH 7.8), 3 mM MgCl_2_, 1 mM EDTA, 1 mM phenylmethylsulfonyl fluoride and 1 mM dithiothreitol, was added. Subsequently, the homogenate was centrifuged at 12,000 × *g* for 10 min at 4 °C. Subsequently, 10 μl of the extract was added to 390 μl assay buffer [50 mM Hepes-Tris (7.8), 3.3 mM MgCl_2_, 0.5 mM D-glucose-6-phosphatedisodium salt and 0.5 mM NADPNa_2_]. The reduction of NADP^ + ^to NADPH was determined as the changing rate of the 340 nm absorbance for the initial 5 min^27^.

To determine the PRK activity of the gametophores at different times during the salt treatment, samples were ground in liquid nitrogen and an extraction buffer (100 mM HEPES-NaOH pH 8.0, 10 mM MgCl2, 0.4 mM EDTA, 1% polyvinylpyrrolidinone, 100 mM Na-ascorbate and 0.1% bovine serum albumin at 4 °C) was added. The enzymes initial activity was measured in a solution containing 30 mM HEPES-NaOH pH 8.0, 10 mM MgCl_2_, 2 mM ATP, 2 mM phosphoenolpyruvate, 1 mM ribose 5-phosphate (R5P), 0.3 mM NADH, and 2 U ml^−1^ of lactate dehydrogenase, pyruvate kinase and ribose phosphate isomerase. The reaction was initiated by adding the extract’s supernatant^42^.

### Determination of NADPH and RNA content

The NADPH content was determined according to Matsumura and Miyachi^43^. An equal wet weight of the gametophores were ground and then transferred to NaOH (0.1 M). The suspensions were kept at 100 °C for 2 min, then cooled to 0 °C and centrifuged (10,000 × *g*, 4 °C). NADPH was extracted into the supernatant obtained after the alkaline treatment. The alkaline extract was neutralized by adding an equivalent amount of HCl, followed by the addition of 0.1 ml of a solution containing 40 mM EDTA, 4.2 mM 3-(4,5-dimethylthiazolyl-2)-2,5-diphenyltetrazolium, 16.6 mM phenazine ethosulfate and 0.05 ml of G6P. After adjusting the total volume to 1 ml by adding H_2_O, the test tubes were kept at 37 °C for 5 min. The reaction was started by adding 0.02 ml of G6P dehydrogenase. After the proper reaction time (30–60 min), the absorbency at 570 nm was measured. The concentration of NADPH in each extract was determined based on the standard curves.

The RNA contents of the gametophores with equal wet weight at different times during salt treatments were extracted using a Total RNA Kit (OMEGA), and the concentrations were measured using a NanoDrop 1000 Spectrophotometer (Thermo) and the purity of the isolated RNA from different conditions was determined through the electrophoresis (Supplementary Figure S2).

### Analysis of starch and soluble sugar contents

The starch and soluble sugar contents were determined according to the methods described by Sánchez, *et al.*^44^ and Brányiková, *et al.*^45^, respectively, with minor modifications. An equal wet weight of the gametophores at different time during salt treatments were frozen in liquid nitrogen and ground into powder. Soluble sugar was extracted using 8 ml of 80% ethanol at 68 °C for 15 min and centrifuged (10,000 × *g*, room temperature). This step was repeated three times. The supernatants were combined and volatilized in an 85 °C water bath to a volume of 2–3 ml. Distilled water was then added to a final total volume of 10 ml. For the total hydrolysis of starch, 3.3 ml of 30% perchloric acid was added to the sediment, stirred for 15 min and centrifuged (10,000 × *g*, room temperature). This step was also repeated three times. The extracts were combined, and perchloric acid was then added to a final volume of 10 ml. Then, 0.1 ml of the soluble sugar and starch extracts were cooled to 0 °C, and 1 ml of 0.2% anthrone solution [0.2 g of anthrone in 100 ml of 72% (v/v) H_2_SO_4_] was added and blended quickly. The mixtures were kept in a water bath at 100 °C for 8 min, cooled to 20 °C, and the 625 nm absorbance levels were measured. A standard curve was created simultaneously using glucose. The soluble sugar contents were determined according to the calibration curve, while the starch contents were obtained by multiplying the measured values by 0.9.

### Statistical analyses

The results were expressed as the mean values of five independent experiments ± standard deviation (SD). Data were used for statistical analysis via one-way analysis of variance (ANOVA) using the SPSS 18.0 statistical software. For the post-hoc analysis, Tukey test was used at an α = 0.05 significance level.

## Additional Information

**How to cite this article**: Gao, S. *et al.* G6PDH activity highlights the operation of the cyclic electron flow around PSI in *Physcomitrella patens* during salt stress. *Sci. Rep.* **6**, 21245; doi: 10.1038/srep21245 (2016).

## Supplementary Material

Supplementary Information
