# Supplementary material for: G6PDH activity highlights the operation of the cyclic electron flow around PSI in Physcomitrella patens during salt stress
Source: Sci Rep. 2016 Feb 18;6:21245. doi: 10.1038/srep21245 (PMC4758081; doi:10.1038/srep21245)
Supplement: Supplementary Information [file srep21245-s1.pdf]

G6PDH activity highlights the operation of the cyclic electron flow around PSI in *Physcomitrella patens* during salt stress

Shan Gao, Zhenbing Zheng, Li Huan and Guangce Wang

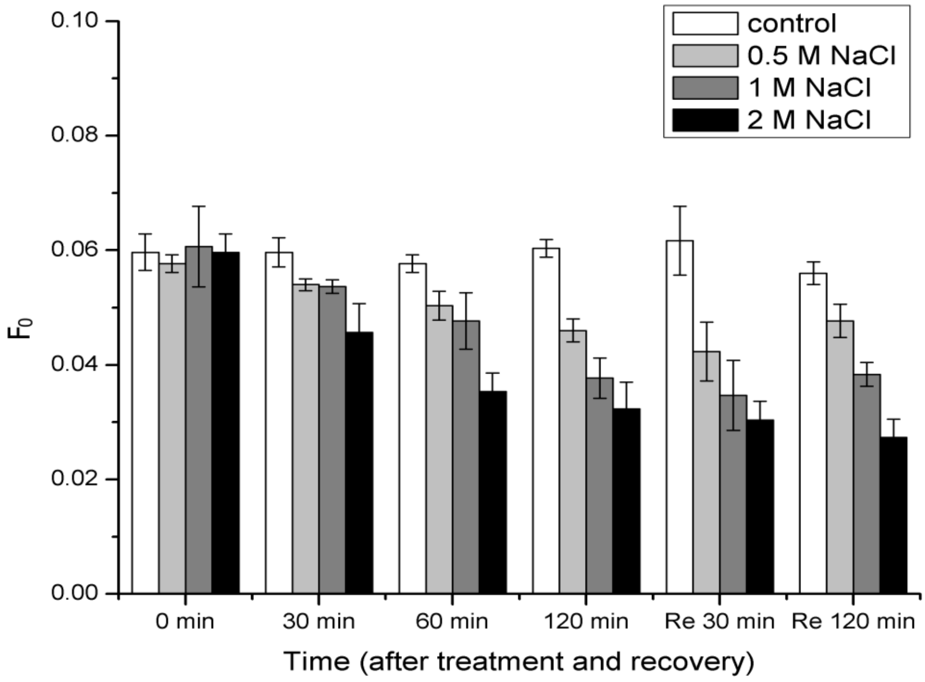

Figure S1. Changes in F_0 in *P. patens* during treatments with control (normal liquid medium), 0.5 M, 1 M and 2 M NaCl solutions and after recovering in normal liquid medium. Re is the abbreviation of recovery. Data shown are the means of five independent experiments (±SD)

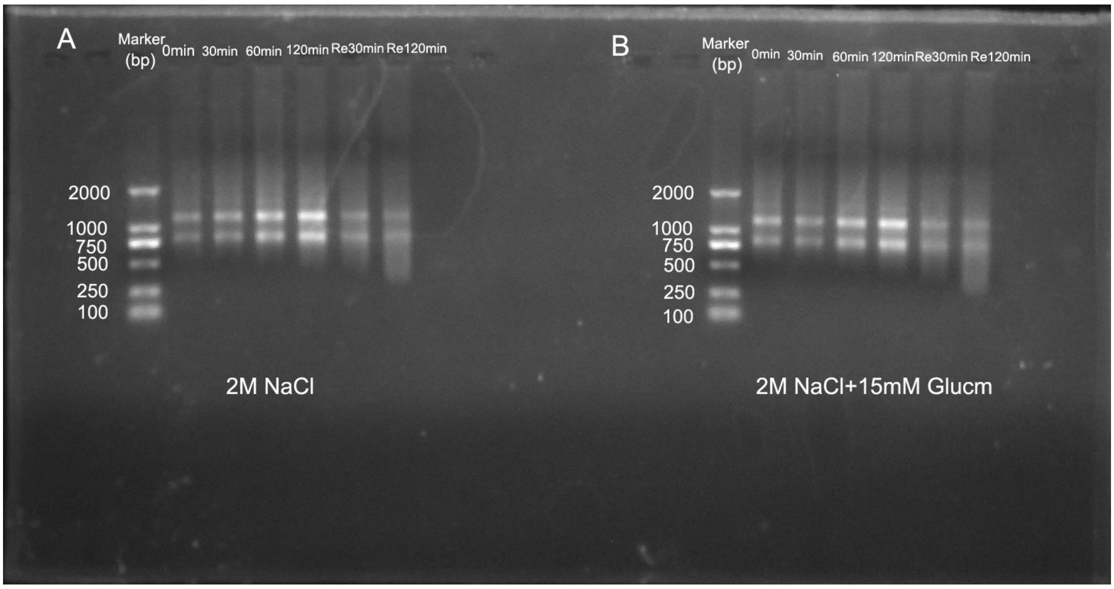

Figure S2. The gel image of isolated RNA from different conditions. (A) 2M NaCl.(B) 2M NaCl containing 15mM Glucm.
